# Auxora vs. placebo for the treatment of patients with severe COVID-19 pneumonia: a randomized-controlled clinical trial

**DOI:** 10.1186/s13054-022-03964-8

**Published:** 2022-04-08

**Authors:** Charles Bruen, Mukhtar Al-Saadi, Edward A. Michelson, Maged Tanios, Raul Mendoza-Ayala, Joseph Miller, Jeffrey Zhang, Kenneth Stauderman, Sudarshan Hebbar, Peter C. Hou

**Affiliations:** 1 Regions Hospital, Health Partners, St. Paul, MN USA; 2 Houston Methodist Hospital, Houston, TX USA; 3 Department of Emergency Medicine, Texas Tech University Health Sciences Center, El Paso, TX USA; 4 MemorialCare Long Beach Medical Center, Long Beach, CA USA; 5 Aurora BayCare Medical Center, Green Bay, WI USA; 6 Henry Ford Hospital System, Detroit, MI USA; 7 Princeton Pharmatech, Princeton, NJ USA; 8 CalciMedica, Inc, 505 Coast Blvd. South Suite 307, La Jolla, CA 92037 USA; 9 Brigham and Women’s Hospital, Harvard Medical School, Boston, MA USA

## Abstract

**Background:**

Calcium release-activated calcium (CRAC) channel inhibitors block proinflammatory cytokine release, preserve endothelial integrity and may effectively treat patients with severe COVID-19 pneumonia.

**Methods:**

CARDEA was a phase 2, randomized, double-blind, placebo-controlled trial evaluating the addition of Auxora, a CRAC channel inhibitor, to corticosteroids and standard of care in adults with severe COVID-19 pneumonia. Eligible patients were adults with ≥ 1 symptom consistent with COVID-19 infection, a diagnosis of COVID-19 confirmed by laboratory testing using polymerase chain reaction or other assay, and pneumonia documented by chest imaging. Patients were also required to be receiving oxygen therapy using either a high flow or low flow nasal cannula at the time of enrolment and have at the time of enrollment a baseline imputed PaO_2_/FiO_2_ ratio > 75 and ≤ 300. The PaO_2_/FiO_2_ was imputed from a SpO_2_/FiO_2_ determine by pulse oximetry using a non-linear equation. Patients could not be receiving either non-invasive or invasive mechanical ventilation at the time of enrolment. The primary endpoint was time to recovery through Day 60, with secondary endpoints of all-cause mortality at Day 60 and Day 30. Due to declining rates of COVID-19 hospitalizations and utilization of standard of care medications prohibited by regulatory guidance, the trial was stopped early.

**Results:**

The pre-specified efficacy set consisted of the 261 patients with a baseline imputed PaO_2_/FiO_2_≤ 200 with 130 and 131 in the Auxora and placebo groups, respectively. Time to recovery was 7 vs. 10 days (*P* = 0.0979) for patients who received Auxora vs. placebo, respectively. The all-cause mortality rate at Day 60 was 13.8% with Auxora vs. 20.6% with placebo (*P* = 0.1449); Day 30 all-cause mortality was 7.7% and 17.6%, respectively (*P* = 0.0165). Similar trends were noted in all randomized patients, patients on high flow nasal cannula at baseline or those with a baseline imputed PaO_2_/FiO_2_ ≤ 100. Serious adverse events (SAEs) were less frequent in patients treated with Auxora vs. placebo and occurred in 34 patients (24.1%) receiving Auxora and 49 (35.0%) receiving placebo *(P* = 0.0616). The most common SAEs were respiratory failure, acute respiratory distress syndrome, and pneumonia.

**Conclusions:**

Auxora was safe and well tolerated with strong signals in both time to recovery and all-cause mortality through Day 60 in patients with severe COVID-19 pneumonia. Further studies of Auxora in patients with severe COVID-19 pneumonia are warranted.

*Trial registration* NCT04345614.

**Supplementary Information:**

The online version contains supplementary material available at 10.1186/s13054-022-03964-8.

## Introduction

The COVID-19 pandemic has caused nearly 5.5 million deaths worldwide and more than 820,000 deaths in the US [1]. Although most cases are asymptomatic or mild, up to 20% of patients progress to develop severe pneumonia, requiring hospitalization and intensive care, with mortality rates near 30% in high-risk groups [2–6]. In the US alone, more than 2.6 million patients with COVID-19 have been hospitalized [7]. To address this global health crisis, antiviral treatments have been utilized to decrease the time to recovery and immunomodulatory therapies have been administered as they have demonstrated some efficacy at reducing mortality among hospitalized patients but additional novel therapeutics are urgently needed [8–10].

In patients with severe COVID-19 pneumonia, broad dysregulated immune responses have been identified with patients showing elevations in a range of proinflammatory cytokines [11–18]. These pathophysiologic events suggest that treatments with broad-based immunomodulatory effects may be more effective in treating COVID-19 pneumonia than those targeting specific immune pathways to prevent disease progression [11–19]. One such potential treatment is Auxora, a calcium release-activated calcium (CRAC) channel inhibitor. CRAC channel inhibition by the active ingredient in Auxora, CM4620, has been shown to block the release of multiple pro-inflammatory cytokines, including interleukin (IL)-6 [20]. Further, in a Phase 2 open-label study in patients with acute pancreatitis with accompanying systemic inflammatory response syndrome (SIRS) and hypoxemia, it was noted that Auxora rapidly lowered IL-6 levels in those patients presenting with levels > 150 pg/mL [21]. The reduction in IL-6 supported the start of an open-label study of Auxora in patients with severe COVID-19 pneumonia in the spring of 2020. The initial open-label trial showed Auxora was safe and reduced the occurrence of a composite of death and need for invasive mechanical ventilation [22]. Given these initial results, a randomized, placebo-controlled trial was initiated to test the hypothesis that the inhibition of CRAC channels by Auxora may effectively treat patients with severe COVID-19 pneumonia.

## Methods

### Trial design and oversight

CARDEA was a phase 2, randomized, double-blind, placebo-controlled trial that tested the addition of Auxora to corticosteroids and standard of care in patients with severe COVID-19 pneumonia (ClinicalTrials.gov identifier, NCT04345614). The study of Auxora in patients with severe COVID-19 pneumonia was initially conducted as an open-label study that started enrollment on April 8, 2020. The FDA provided guidance on May 12, 2020, to limit further enrollment under the open-label design and transition to a randomized, double-blind, placebo-controlled trial. As such, the open-label study was terminated on May 13, 2020 and the results were published [22].

CARDEA was initially designed to enroll 400 patients with two specified groups that were to be stratified equally across the treatment arms: 80 patients with a baseline imputed PaO_2_/FiO_2_ ratio of > 200 and 320 patients with a baseline imputed PaO_2_/FiO_2_ ratio of ≤ 200. The PaO_2_/FiO_2_ was imputed from a SpO_2_/FiO_2_ using a non-linear equation. The SpO_2_ was obtained using pulse oximetry. The FiO_2_ was read from the controlled oxygen source in patients requiring high flow nasal cannula. For patients on an uncontrolled oxygen source, a conversion table was provided to all sites to estimate the FiO_2_ based on the method of oxygen delivery and oxygen flow rate [23]. The baseline imputed PaO_2_/FiO_2_ was the worst value in the 24 h prior to screening.

It had been noted in the open-label study that patients with a baseline imputed PaO_2_/FiO_2_ > 200 had neither required invasive mechanical ventilation nor died so their enrollment in CARDEA was to confirm this observation [22]. After the first 23 patients with a baseline imputed PaO_2_/FiO_2_ > 200 were randomized, a blinded analysis confirmed this observation. As a result, further enrollment of this patient subgroup was halted following the first IDMC review to avoid impacting efficacy signals from the study. From that point forward, only patients with a baseline imputed PaO_2_/FiO_2_ ≤ 200 were randomized into the study with the enrollment goal of 320 patients in this group being unchanged. The study was terminated, however, after 261 patients with a baseline imputed PaO_2_/FiO_2_ ratio ≤ 200 were randomized based on declining rates of US COVID-19 hospitalizations in the spring of 2021 and the more frequent use of tocilizumab in CARDEA candidate patients at many trial sites following recommendations by the National Institutes of Health’s COVID-19 Treatment Guidelines Panel [24]. The use of tocilizumab in combination with Auxora had been prohibited by regulatory guidance.

In CARDEA, patients were randomized 1:1 to Auxora plus standard of care or placebo plus standard of care. Participants, investigators, study teams, and the sponsor were all blinded to study drug assignment. Randomization was stratified by the baseline imputed PaO_2_/FiO_2_ ratio of > 200 vs ≤ 200 through a central, concealed, web-based, automated system. An independent statistician created the randomization schedule with stratified block randomization method using SAS proc plan procedure. Within each stratum, the treatment codes were assigned at a 1:1 ratio of Auxora and placebo with the block size of 4.

Auxora was administered by a 4-h IV infusion at 2.0 mg/kg (1.25 mL/kg) at 0-h and 1.6 mg/kg (1 mL/kg) at 24 and 48 h. Placebo was a matching formulation without the active pharmaceutical ingredient and was also dosed as a 4-h IV infusion at 1.25 mL/kg at 0-h and 1 mL/kg at 24 and 48 h. Patients were assessed for recovery and mortality using an ordinal scale in a standardized manner as described in the electronic case report form. The initial assessments occurred immediately before each infusion. Seventy-two hours after the start of the first infusion, patient assessment occurred every 24 h (± 4 h) until 240 h and then continued every 48 h until Day 30 or discharge. Patients discharged before Day 25 were contacted at Day 30 (± 5 days). All patients were again assessed Day 60 (± 5 days); patients who remained in the hospital after Day 30 were assessed by review of hospital records and those who had been discharged were contacted by telephone. Public information (e.g., death reports, governmental information) was used by sites to ascertain Day 60 mortality status in patients who refused direct contact after discharge or had withdrawn from the trial.

All patients were required to receive dexamethasone or equivalent dose of another corticosteroid as well as pharmacological prophylaxis against development of venous thromboembolic disease. Remdesivir use was recommended for all patients, and convalescent plasma administration was allowed according to local standard of care. Other immunomodulators for the treatment of COVID-19 pneumonia, including tocilizumab and JAK inhibitors, were prohibited due to regulatory guidance.

An institutional review board at each site approved the trial protocol. Informed consent was obtained from the patient or the patient’s legally authorized representative if the patient was unable to provide consent. The trial was conducted in accordance with Good Clinical Practice guidelines and the principles of the Declaration of Helsinki, and was sponsored by CalciMedica, Inc (La Jolla, CA). An independent data monitoring committee (IDMC) provided trial oversight. Operational support was provided by Bionical-Emas (Paulsboro, NJ) and Princeton Pharmatech (San Francisco, CA) performed the statistical analyses. All authors vouch for the accuracy and completeness of the data and for the fidelity of the trial adherence to the protocol.

The IDMC first reviewed unblinded safety data once 57 patients were randomized, then again after 70 patients with a baseline imputed PaO_2_/FiO_2_ ≤ 200 completed 60 days of the trial, and finally after randomization of 209 patients with a baseline imputed PaO_2_/FiO_2_ ≤ 200. The IDMC also performed an interim sample size re-estimation based on the recovery rate ratio after 70 patients with a baseline imputed PaO_2_/FiO_2_ ≤ 200 reached Day 60.

### Patient population

Eligible patients were adults with ≥ 1 symptom consistent with COVID-19 infection, a diagnosis of COVID-19 confirmed by laboratory testing using polymerase chain reaction or other assay, and pneumonia documented by chest imaging. Patients were also required to be receiving oxygen therapy using either a high flow (HFNC) or low flow nasal cannula and have at the time of enrolment a baseline imputed PaO_2_/FiO_2_ ratio > 75 and ≤ 300. Patients could not be receiving either non-invasive or invasive mechanical ventilation at the time of enrolment. Full inclusion and exclusion criteria are available in the Additional file 1: Appendix.

### Outcomes

The primary endpoint was time to recovery through Day 60, defined as meeting the criteria for category 6 (Hospitalized, not requiring supplemental oxygen or ongoing medical care), category 7 (Discharged, requiring supplemental oxygen), or category 8 (Discharged, not requiring supplemental oxygen) using an 8-point ordinal scale. The key secondary endpoint of all-cause mortality at Day 60 was requested by regulatory guidance. Additional secondary endpoints evaluated in the efficacy set included all-cause mortality at Day 30, the proportion of patients requiring invasive mechanical ventilation or death through Day 60, the proportion of patients requiring invasive mechanical ventilation through Day 60, and differences in outcomes measured by the 8-point ordinal scale through Day 60. Safety endpoints included the occurrence and severity of treatment-emergent adverse events (TEAEs) and serious AEs (SAEs).

The primary and key secondary endpoints were also evaluated in pre-specified subgroups of patients who required oxygen therapy via either HFNC or low flow nasal cannula at baseline or patients having a baseline imputed PaO_2_/FiO_2_ ≤ 100 or 101–200 at baseline, and in all randomized patients. The safety endpoints were evaluated in all patients who received study drug, including those with a baseline imputed PaO_2_/FiO_2_ > 200.

### Statistical analysis

The efficacy set was pre-specified, consisting of those patients with a baseline imputed PaO_2_/FiO_2_ ≤ 200. A two-group log-rank test with a 0.05 two-sided significance level would have 90% power to detect a difference in the recovery rate ratio of approximately 1.49 in the 320 patients with a baseline imputed PaO_2_/FiO_2_ ≤ 200 who were randomized 1:1 to Auxora or placebo. The Sponsor elected to not change the sample size after the IDMC performed the sample size re-estimation. All supplemental analyses of the primary and first secondary endpoints were also performed in a set of all randomized patients.

Time to recovery through Day 60 in the efficacy set was compared between the Auxora and placebo groups using log-rank test stratified by baseline imputed PaO_2_/FiO_2_ ≤ 100 vs. 101–200 and displayed using a Kaplan–Meier estimate. Patients were censored at the last ordinal scale assessment if no recovery event was observed during the study and if they had recovered, been discharged, but Day 60 recovery status was not obtained.

All-cause mortality at Day 60 in the efficacy set was compared between the Auxora and placebo groups using a Cochran-Mantel- Haenszel test stratified by the baseline imputed PaO_2_/FiO_2_ ≤ 100 vs. 101–200. In addition, a sensitivity analysis was performed that estimated the 60-day death rate with hypothesis testing based on the Kaplan–Meier estimates and standard errors estimated by Greenwood formula using the log–log transformation of the survival function stratified by the baseline imputed PaO_2_/FiO_2_ of ≤ 100 vs. 101–200.

To protect the trial level type 1 error rate at *α* = 5% (two sided) between the primary endpoint analysis and the key secondary endpoint analysis, the Benjamini and Hochberg testing strategy was used as test statistics of time to recovery and all-cause mortality at Day 60 were positively correlated.

### Role of funding source

The funder of the study had primary responsibility for the study design, protocol development, study monitoring, data management and interpretation, and statistical analyses. The funder also contributed to the drafting of the manuscript and decision to submit.

## Results

### Patients

**Fig. 1:**
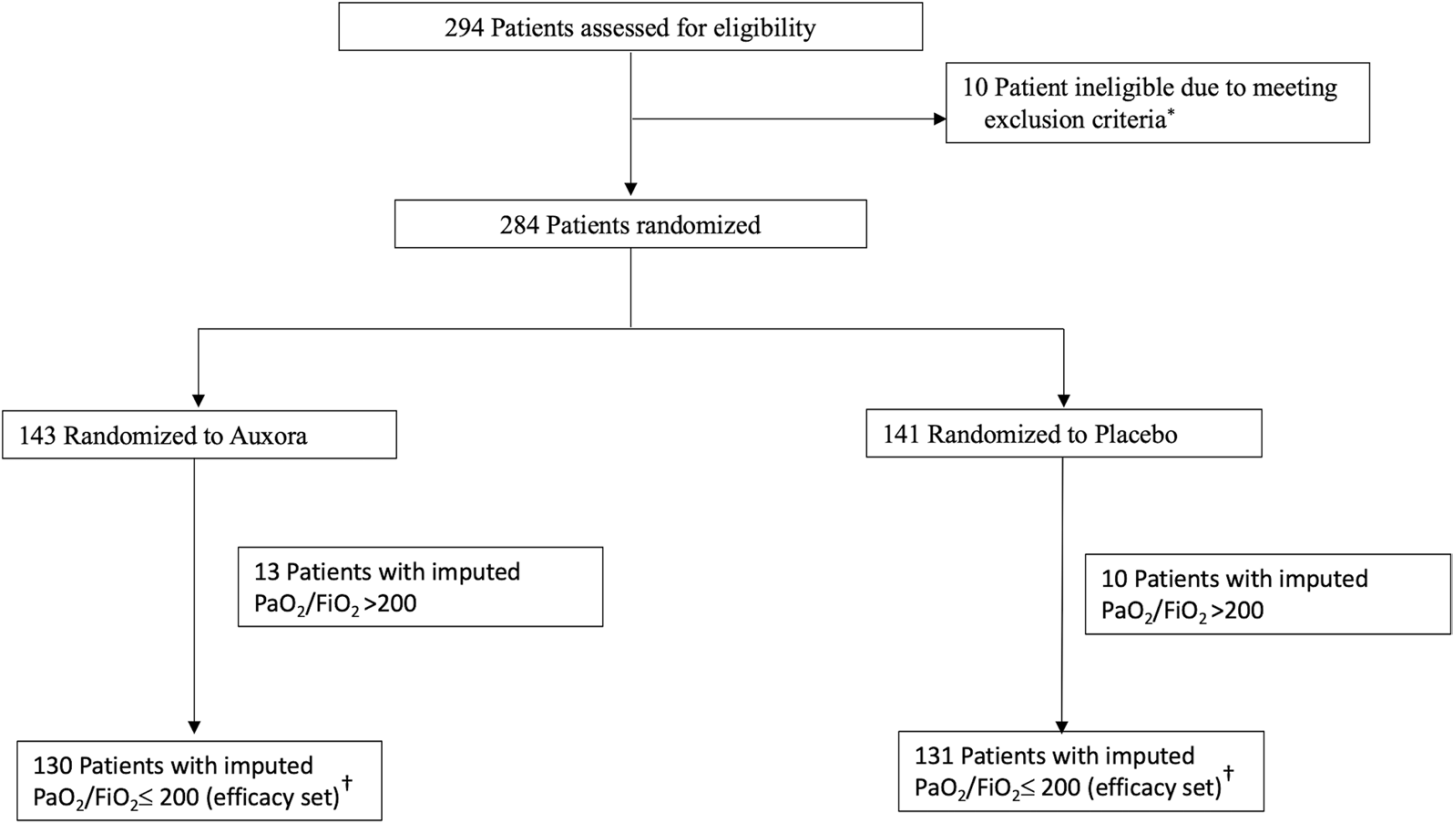
Patient Enrolment and Randomization. *Reasons for screen failure included PaO_2_/FiO_2_ ≤ 75 (*n* = 3), at least 1 of the following signs at Screening or noted in the 24 h before Screening: SpO2 < 92% on room air; PaO_2_/FiO_2_ = 300 when receiving low flow supplemental oxygen (*n* = 3), do not intubate order (*n* = 2), prohibited medication (*n* = 1), history of organ or hematologic transplant, HIV, Active hepatitis B, or hepatitis C infection (*n* = 1); ^†^One patient in the Auxora arm and one patient in the placebo arm who had a baseline imputed PaO_2_/FiO_2_ ≤ 200 at baseline did not receive any doses

Patient enrollment occurred from September 8, 2020 to May 24, 2021. A total of 284 patients were randomized across 17 US centers, 143 to Auxora and 141 to placebo (Fig. 1), and 281 patients received at least one dose of study drug. The efficacy set consisted of 261 patients with a baseline imputed PaO_2_/FiO_2_ ≤ 200 with 130 in Auxora and 131 in placebo groups (Fig. 1). One patient was lost to follow up. In total, Day 60 mortality status was documented in 283 and Day 60 recovery status as determined by the ordinal scale was documented in 275 of the 284 patients randomized in the study.

All patients (100%) in the efficacy set received corticosteroids (85.8%, dexamethasone), 75.9% received remdesivir, and 99.6% received anticoagulation (93.1%, enoxaparin [dosed for venous thromboembolic disease prophylaxis]; Additional file 1: Table S1). Tocilizumab, while a prohibited medication, was administered to 8 patients after randomization, and 6 were determined to have received placebo after unblinding.

Baseline characteristics were well balanced between the Auxora and placebo groups in the efficacy set (Table 1) and among all randomized patients (Additional file 1: Table S2). The mean age across both treatment arms was 60 years; 67.4% were male, and 39.5% were Hispanic or Latino; there was a higher percentage of men in the placebo group. The mean time from symptom onset to randomization was 12 days, and 62.5% required oxygen therapy via HFNC at baseline; 44.8% of patients had a baseline imputed PaO_2_/FiO_2_ ≤ 100 at baseline.

**Table 1:** Baseline characteristics in patients with a baseline imputed PaO_2_/FiO_2_ ≤ 200

	Placebo (*n* = 131)	Auxora (*n* = 130)	Total (*N* = 261)
Male, *n* (%)	92 (70.2%)	84 (64.6%)	176 (67.4%)
**Race**			
White, *n* (%)	98 (74.8%)	85 (65.4%)	183 (70.1%)
Black, *n* (%)	12 (9.2%)	19 (14.6%)	31 (11.9%)
Asian	5 (3.8%)	9 (6.9%)	14 (5.4%)
Other/multiple*	16 (12.2%)	16 (12.3%)	32 (12.3%)
Hispanic, *n* (%)	58 (44.3%)	45 (34.6%)	103 (39.5%)
Mean age, years (SD)	60.4 (12.3)	59.4 (12.1)	59.9 (12.2)
65+ years of age, *n* (%)	47 (35.9%)	45 (34.6%)	92 (35.2%)
Mean BMI, kg/m^2^ (SD)	32.0 (7.0)	32.8 (8.8)	32.4 (8.0)
Mean time from symptom onset, days (SD)	12.0 (5.9)	12.2 (5.8)	12.1 (5.8)
Median time from hospitalization to randomization, days	3.0	3.0	3.0
HFNC use, *n* (%)	82 (62.6%)	81 (62.3%)	163 (62.5%)
Mean baseline imputed PaO_2_/FiO_2_ value^†^ (SD)	105.1 (32.8)	109.7 (36.8)	107.4 (34.8)
Baseline imputed PaO_2_/FiO_2_ ≤ 100, *n* (%)	58 (44.3%)	59 (45.4%)	117 (44.8%)
Baseline imputed PaO_2_/FiO_2_ 101–200, *n* (%)	73 (55.7%)	71 (54.6%)	144 (55.2%)
Mean CRP, mg/L (SD)	92.5 (67.6)	93.1 (71.2)	92.8 (69.2)
Mean ferritin, ng/mL (SD)	1050 (869)	1027 (907)	1039 (886)
Hypertension, *n* (%)	80 (61.1%)	84 (64.6%)	164 (62.8%)
Diabetes, *n* (%)	57 (43.5%)	52 (40.0%)	109 (41.8%)
Hyperlipidemia, *n* (%)	51 (38.9%)	50 (38.5%)	101 (38.7%)
Former Smoker, *n* (%)	34 (26.0%)	39 (30.0%)	73 (28.0%)

*Other include Native Hawaiian or other Pacific Islander. One participant in the Auxora group was missing race at baseline

^†^Worst value in the 24 h prior to Screening. Patients with a baseline imputed PaO_2_/FiO_2_ > 200 were excluded from the efficacy set analysis

*BMI* body mass index, *CRP* C-reactive protein, *HFNC* high flow nasal cannula, *SD* standard deviation

### Time to recovery

The median time to recovery was 7 days (95% CI, 6.0, 9.0) and 10 days (95% CI, 7.0, 14.0; *P* = 0.0979) for patients in the Auxora and placebo groups, respectively (Table 2). In the subgroups of patients who required oxygen therapy via HFNC at baseline or had a baseline imputed PaO_2_/FiO_2_ ≤ 100 at baseline, patients in the Auxora group had a faster time to recovery, compared to the placebo group (Table 3). Similar results were noted when all randomized patients were analyzed (Additional file 1: Table S3).

**Table 2:** Primary and secondary endpoints in patients with a baseline imputed PaO_2_/FiO_2_ ≤ 200

	Placebo (*n* = 131)	Auxora (*n* = 130)	Difference (95% CI)	*P* value
**Primary endpoint**				
Median time to recovery, days (95% CI)	10.0 (7.0, 14.0)	7.0 (6.0, 9.0)		0.0979
**Secondary endpoints**				
All-cause mortality at Day 60, *n* (%)	27 (20.6%)	18 (13.8%)	− 6.75 (− 15.75, 2.24)	0.1449
All-cause mortality at Day 30, *n* (%)	23 (17.6%)	10 (7.7%)	− 9.86 (− 17.80, − 1.93)	0.0165
Invasive Mechanical Ventilation, Proportion of Patients Day 60 (95% CI)	0.28 (0.21, 0.37)	0.19 (0.13, 0.28)		0.1882
Invasive Mechanical ventilation or death, proportion of patients Day 60 (95% CI)	0.31 (0.24, 0.39)	0.23 (0.17, 0.31)		0.2994

Definition of Recovery by Ordinal Scale: 6 Hospitalized, not requiring supplemental oxygen or ongoing medical care; 7 Discharged, requiring supplemental oxygen; 8 Discharged, not requiring supplemental oxygen. Analysis of time to recovery through Day 60 in the efficacy set used log-rank test stratified by the baseline imputed PaO_2_/FiO_2_ ≤ 100 and 101–200; Analysis of all-cause mortality in the efficacy set used Cochran-Mantel–Haenszel test stratified by the baseline imputed PaO_2_/FiO_2_ ≤ 100 and 101–200. Patients with a baseline imputed PaO_2_/FiO_2_ > 200 were excluded from the efficacy set analysis

**Table 3:** Time to recovery and all-cause mortality by oxygen delivery mode and baseline imputed PaO_2_/FiO_2_ at baseline

Oxygen delivery at baseline				
HFNC				
	Placebo (*n* = 82)	Auxora (*n* = 81)	Difference (95% CI)	*P* value
Median time to recovery, days (95% CI)	17.0 (8.0, 30.0)	9.0 (7.0, 13.0)		0.1079
All-Cause Mortality at Day 60, n (%)	21 (25.6%)	13 (16.0%)	−9.36 (−21.79, 3.07)	0.1436

Low flow oxygen				
	Placebo (*n* = 49)	Auxora (*n* = 49)	Difference (95% CI)	*P* value
Median time to recovery, days (95% CI)	7.0 (5.0, 9.0)	5.0 (4.0, 6.0)		0.4195
All-Cause Mortality at Day 60, *n* (%)	6 (12.2%)	5 (10.2%)	−2.04 (−14.53, 10.45)	0.7490

Baseline imputed PaO_2_/FiO_2_ at baseline				
** ≤ 100**				
	Placebo (*n* = 58)	Auxora (*n* = 59)	Difference (95% CI)	*P* value
Median time to recovery, days (95% CI)	23.0 (11.0, 70.0)	11.5 (8.0, 23.0)		0.1040
All-Cause Mortality at Day 60, *n* (%)	17 (29.3%)	12 (20.3%)	−8.62 (−24.30, 7.06)	0.2837

101–200				
	Placebo (*n* = 73)	Auxora (*n* = 71)	Difference (95% CI)	*P* Value
Median time to recovery, days (95% CI)	7.0 (5.0, 8.0)	6.0 (5.0, 7.0)		0.4156
All-Cause Mortality at Day 60, *n* (%)	10 (13.7%)	6 (8.5%)	−5.25 (−15.45, 4.95)	0.3164

Definition of Recovery by Ordinal Scale: 6 Hospitalized, not requiring supplemental oxygen or ongoing medical care; 7 Discharged, requiring supplemental oxygen; 8 Discharged, not requiring supplemental oxygen. Kaplan–Meier estimate of Days to Recovery with P value based on log-rank test without stratification. Unstratified analysis of mortality using Chi-squared test. HFNC, high flow nasal cannula. Patients with a baseline imputed PaO_2_/FiO_2_ > 200 were excluded from the efficacy set analysis

### All-cause mortality

The all-cause mortality rate at Day 60 was 13.8% (*n* = 18) in patients treated with Auxora and 20.6% (*n* = 27) with placebo (difference −6.75; 95% CI −15.75, 2.24; *P* = 0.1449; Table 2; Additional file 1: Figure S1). The all-cause mortality rate at Day 30 was 7.7% in patients treated with Auxora and 17.6% with placebo (difference −9.86; 95% CI −17.80, −1.83; *P* = 0.0165; Table 2; Additional file 1: Figure S1). Lower mortality rates at Day 60 were observed in subgroups of patients using HFNC at baseline or those with a baseline imputed PaO_2_/FiO_2_ ≤ 100 at baseline (Table 3; Additional file 1: Figures S2, S3) and in all randomized patients (Additional file 1: Table S3). Additional pre-specified subgroup analyses for mortality are noted in Additional file 1: Figure S4.

**Fig. 2:**
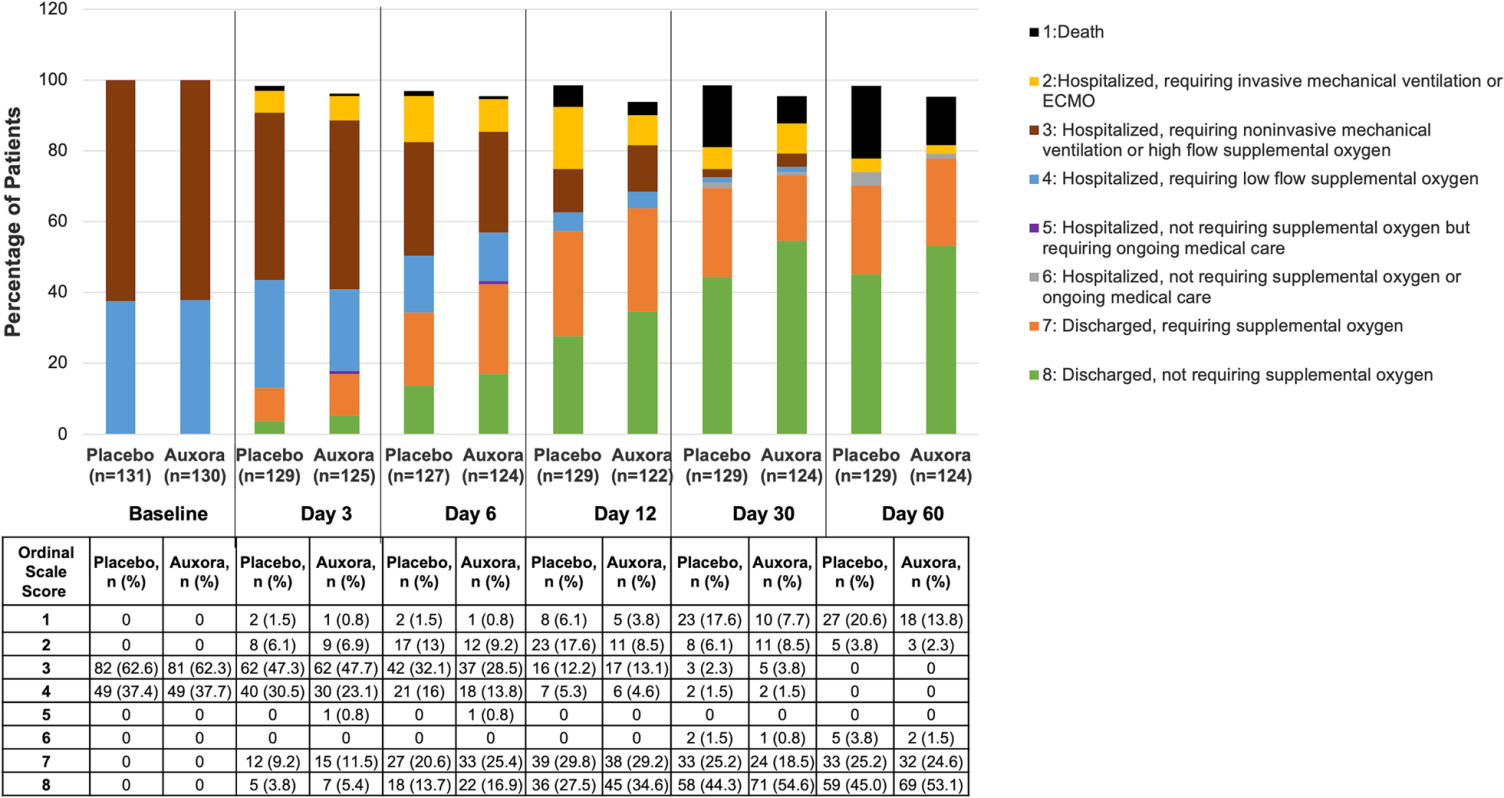
Proportion of Patients with a Baseline Imputed PaO_2_/FiO_2_ ≤ 200 in Each Ordinal Scale Category Over Time. A higher proportion of patients receiving Auxora were discharged, and a lower proportion progressed to invasive mechanical ventilation, ECMO, and death at Day 60 (Odds Ratio, 0.647; 95% CI 0.405, 1.031; *P* = 0.0672) and Day 30 (Odds Ratio, 0.617; 95% CI 0.387, 0.983; *P* = 0.0423). Efficacy outcome measured with the 8-point ordinal scale included recovery rate defined as the first day the patient satisfied criteria 6, 7, or 8 and change in the 8-point ordinal scale over time. The proportions are compared between the two treatment groups using a proportional odds model with a fixed factor of treatment groups. ECMO, Extracorporeal membrane oxygenation

Additional secondary endpoints demonstrated potential benefits with Auxora vs. placebo (Table 2), including a higher proportion of patients receiving Auxora being discharged, and a lower proportion progressing to invasive mechanical ventilation, extracorporeal membrane oxygenation, and death at Day 60 (Odds Ratio, 0.647; 95% CI 0.405, 1.031; *P* = 0.0672) and Day 30 (Odds Ratio, 0.617; 95% CI 0.387, 0.983; *P* = 0.0423; Fig. 2).

### Safety outcomes

In total, 34 patients (24.1%) in the Auxora and 49 (35.0%) in the placebo groups experienced SAEs (*P* = 0.0616). The most common SAEs were respiratory failure, acute respiratory distress syndrome (ARDS), and pneumonia (Table 4). Discontinuation due to TEAEs occurred in 3 patients in the Auxora and 5 patients in the placebo groups. The most common TEAEs were respiratory failure, increasing triglycerides, hyperglycemia, and acute kidney injury.

**Table 4:** Safety outcomes in all patients receiving at least one dose of study drug

	Placebo (*n* = 140)	Auxora (*n* = 141)
Discontinuation due to AEs, *n* (%)	5 (3.6%)	3 (2.1%)
Serious adverse events ≥ 4%, *n* (%)		
Respiratory failure	26 (18.6%)	22 (15.6%)
ARDS	11 (7.9%)	7 (5.0%)
Pneumonia	7 (5.0%)	6 (4.3%)
Cardiac arrest	6 (4.3%)	6 (4.3%)
Septic shock	8 (5.7%)	2 (1.4%)
Most common treatment-emergent adverse events ≥ 4%, *n* (%)		
Respiratory failure	26 (18.6%)	22 (15.6%)
Blood triglycerides increased	5 (3.6%)	16 (11.3%)
Hypertriglyceridemia	4 (2.9%)	2 (1.4%)
Hyperglycemia	11 (7.9%)	11 (7.8%)
Acute kidney injury	16 (11.4%)	10 (7.1%)
Increased transaminases	5 (3.6%)	8 (5.7%)
Liver function test increased	1 (0.7%)	5 (3.5%)
ARDS	11 (7.9%)	7 (5.0%)
DVT	7 (5.0%)	7 (5.0%)
Pneumonia	7 (5.0%)	7 (5.0%)
Pneumothorax	6 (4.3%)	7 (5.0%)
Pneumomediastinum	2 (1.4%)	6 (4.3%)
Hypoxia	7 (5.0%)	6 (4.3%)
Cardiac arrest	6 (4.3%)	6 (4.3%)
Hyperkalemia	6 (4.3%)	4 (2.8%)
Anemia	9 (6.4%)	3 (2.1%)
Septic Shock	13 (9.3%)	2 (1.4%)

*AEs* adverse events, *ARDS* acute respiratory distress syndrome, *DVT* deep vein thrombosis

## Discussion

Auxora was initially studied in patients with acute pancreatitis and accompanying SIRS and hypoxemia [21]. In this study, it was noted that Auxora decreased IL-6 levels in patients presenting with IL-6 ≥ 150 pg/mL, including 2 patients with values > 1000 pg/mL [21]. This result was consistent with in vitro effects of Auxora on cytokine release in human lymphocytes [20]. Based on these findings and the idea that COVID-19 pneumonia involved dysregulated immune and endothelial responses, it was hypothesized that Auxora may be effective in treating patients with severe COVID-19 pneumonia. Mortality and biomarker results from an initial, open-label study of Auxora in patients with severe COVID-19 pneumonia encouraged the transition to the current randomized, double-blind, placebo-controlled CARDEA trial [22, 25].

Results from the CARDEA trial suggest a potential therapeutic benefit of Auxora in addition to corticosteroids and standard of care in patients with severe COVID-19 pneumonia [22]. While not statistically significant, more patients with a baseline imputed PaO_2_/FiO_2_ ≤ 200 who received Auxora met the primary endpoint of time to recovery through Day 60. In addition, patients who received Auxora had a lower all-cause mortality rate at both Days 60 and 30. Patients, who required oxygen therapy via HFNC at baseline or had a baseline imputed PaO_2_/FiO_2_ ≤ 100 at baseline may have benefited the most from the addition of Auxora to corticosteroids and standard of care. There were similar trends when all randomized patients were analyzed.

Auxora was generally safe and well tolerated. Of note, reported AEs for elevated blood triglyceride levels and liver function tests were increased in patients in the Auxora arm when compared with placebo. None of the episodes of hypertriglyceridemia in the Auxora group were reported as being severe. One case of elevated transaminases in the Auxora group was considered severe and occurred in a patient also receiving remdesivir, simvastatin, and ezetimibe. The increased levels resolved with discontinuation of Auxora.

**Fig. 3:**
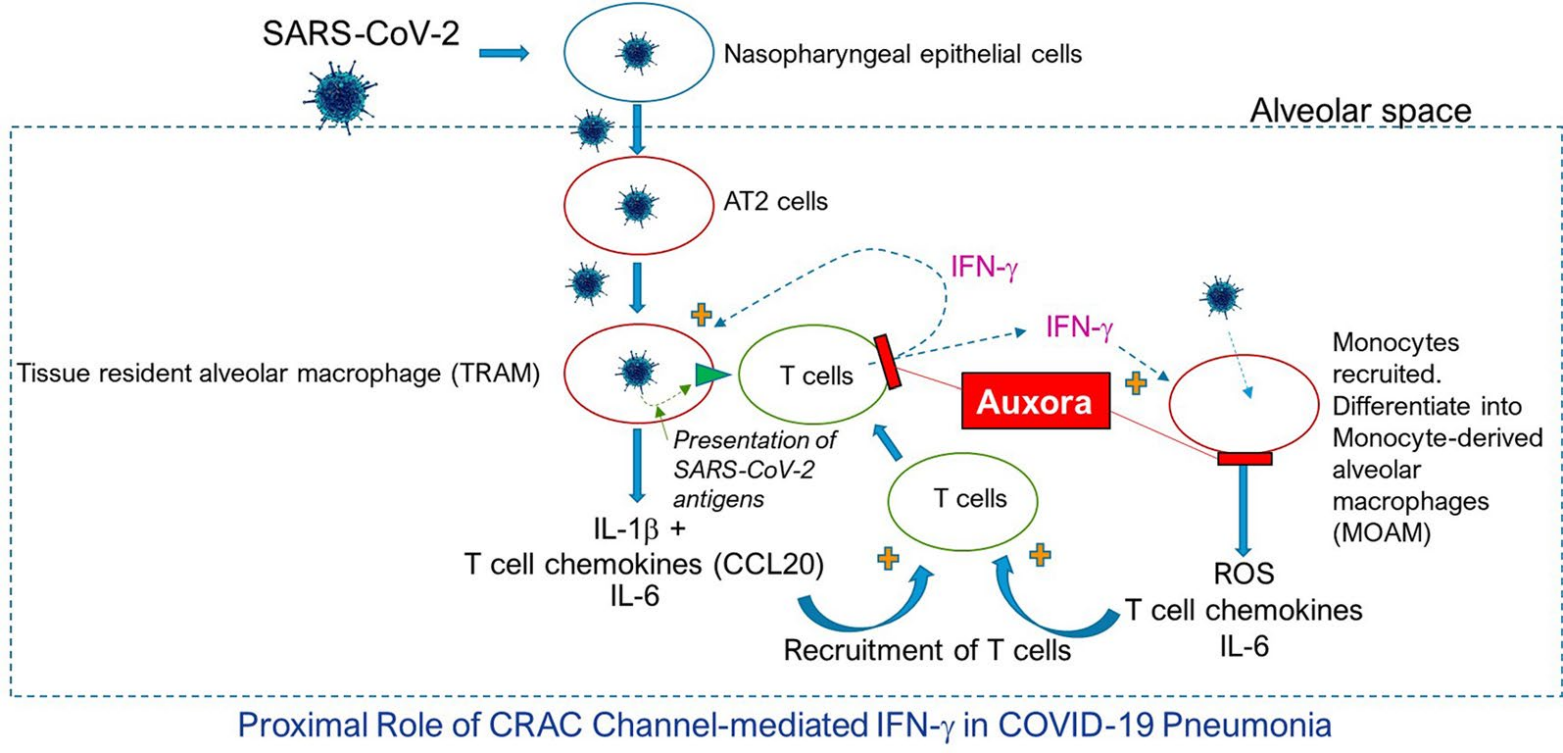
Proximal Role of CRAC Channel-mediated IFN-γ in COVID-19 Pneumonia. Tissue resident alveolar macrophages respond to SARS-CoV-2 infection in the lung by producing T-cell chemoattractants. Arriving T cells produce IFNγ, leading to further alveolar macrophage activation and recruitment of monocyte-derived alveolar macrophages [15, 16]. The feedback loop leads to a rapid increase in proinflammatory cytokines, diffuse alveolar injury, severe endothelialitis, ARDS, and multiorgan dysfunction and failure [14, 17, 18]. Auxora abrogates the release of multiple proinflammatory cytokines from human lymphocytes, including IL-6, IL-17, and IFNγ that are implicated in COVID-19 alveolitis [16, 27]. Adapted from Grant RA, et al. Circuits between infected macrophages and T cells in SARS-CoV-2 pneumonia. *Nature.* 2021;590(7847);635–641. IL, interleukin; IFNγ, interferon-gamma; ROS, reactive oxygen species

CRAC channels have been shown to play important roles in several cell types and pathways linked to COVID-19 pneumonia [26]. These channels are mainly composed of the plasma membrane Ca^2+^ conductance protein Orai1 and the endoplasmic reticulum (ER) Ca^2+^ sensing protein stromal interaction molecule 1 (STIM1) [26]. When Ca^2+^ is released from the ER, the drop in ER luminal Ca^2+^ concentration is sensed by STIM1, which undergoes a conformational change resulting in Orai1 activation and Ca^2+^ entry into the cell [26]. Blockade of CRAC channels with the selective Orai1 CRAC channel inhibitor Auxora abrogates the release of multiple proinflammatory cytokines from human lymphocytes, including IL-6, IL-17, and IFNγ that have been implicated in COVID-19 alveolitis (Fig. 3) [16, 27]. Since the Ca^2+^ entering through CRAC channels in T cells primarily activates the calcineurin/nuclear factor of activated T-cells signal transduction pathway, CRAC channel inhibitors may act cooperatively with standard of care anti-inflammatory drugs such as dexamethasone that work through the NF-kB signal transduction pathway [27–29]. Inhibitors of IL-6, such as tocilizumab, and JAK inhibitors, such as baricitinib, may also work in concert with CRAC channel inhibition, although the safety of these combinations is yet to be studied. Importantly, in addition to effects on the immune system, pathophysiologically-activated CRAC channels have been associated with pulmonary endothelial cell dysfunction and plasma extravasation in animal models of acute lung injury [30, 31]. CRAC channel inhibition in these models protects endothelial cells and reduces inflammation and plasma extravasation [31, 32]. Finally, CRAC channels have been shown to regulate cytokine release from alveolar macrophages, which have been implicated in COVID-19 pneumonia [16, 33]. Thus, inhibition of CRAC channels by Auxora may provide the kind of broad-based approach likely to be effective in treating patients with severe COVID-19 pneumonia (Fig. 3).

Limitations in this study include the early termination of the study that caused the study to be underpowered, as the total number of study patients was reduced from the originally planned 400 to 284. In addition, the studied population is a small percentage of the total number of patients hospitalized with COVID-19, as patients with a baseline imputed PaO_2_/FiO_2_ > 300, with a baseline imputed PaO_2_/FiO_2_ ≤ 75, and patients already receiving noninvasive or invasive mechanical ventilation were not enrolled in the study. Therefore, these results may not extend to a broader population with COVID-19. In addition, there are concerns about the validity of imputing the PaO_2_/FiO_2_ ratio using the non-linear formula in patients with COVID-19 as it can influence the definition of severe pneumonia. Finally, while this study should be considered as proof-of-concept for the use of Auxora in the treatment of patients with severe COVID-19 pneumonia, the added benefit and risk of Auxora used in combination with currently accepted standard of care medications is unknown and will require testing in future clinical trials. In the initial open label study, the sponsor obtained regulatory approval to allow investigators to administer corticosteroids to patients with progressing COVID-19 pneumonia [22]. Other immunomodulatory medications were prohibited. CARDEA initiated enrollment after steroids had become accepted as standard of care for patients hospitalized with COVID-19 pneumonia [24]. While CARDEA was underway, baricitinib plus remdesivir was shown to be superior to remdesivir alone in reducing recovery time in patients on high flow ventilation and non-invasive ventilation [10]. Tocilizumab and sarilumab, medications prohibited in CARDEA by regulatory guidance, were also shown to improve outcomes, including mortality, in critically ill patients with COVID-19 receiving organ support in the intensive care unit [34].

## Conclusions

Mechanistically, CRAC-channel inhibitors, such as Auxora, may have therapeutic efficacy in both hastening recovery and reducing mortality in severe COVID-19 pneumonia, and as such, warrant continued clinical development. Results from this phase 2 trial demonstrated that Auxora was safe and well tolerated with strong signals in both time to recovery and all-cause mortality. These results provide support for a follow-up trial of Auxora in patients with severe COVID-19 pneumonia to confirm benefit when used in combination with current standard of care.

## Supplementary Information

**Additional file 1**. Supplemental appendix.

## Data Availability

The datasets generated and/or analyzed during the current study are not publicly available due to the Clinical Study Report being finalized but will be available from the corresponding author upon request.

## Abbreviations

AEs: Adverse events
ARDS: Acute respiratory distress syndrome
BMI: Body mass index
CRAC: Calcium release-activated calcium channels
CRP: C-reactive protein
DVT: Deep vein thrombosis
ECMO: Extracorporeal membrane oxygenation
ER: Endoplasmic reticulum
HFNC: High flow nasal cannula
IDMC: Independent data monitoring committee
IFNγ: Interferon-gamma
IL: Interleukin
ROS: Reactive oxygen species
SAEs: Serious adverse events
SARS-CoV-2: Severe acute respiratory syndrome coronavirus 2
SIRS: Systemic inflammatory response syndrome
STIM1: Stromal interaction molecule 1
TEAEs: Treatment-emergent adverse events
