# Supplementary material for: Auxora vs. placebo for the treatment of patients with severe COVID-19 pneumonia: a randomized-controlled clinical trial
Source: Crit Care. 2022 Apr 8;26:101. doi: 10.1186/s13054-022-03964-8 (PMC8992417; doi:10.1186/s13054-022-03964-8)
Supplement: Supplementary file 1 — Additional file 1. Supplemental appendix. [file 13054_2022_3964_MOESM1_ESM.docx]

**Supplemental appendix**

**Auxora vs. placebo for the treatment of patients with severe COVID-19 pneumonia: a randomized clinical trial**

Table of Contents

[Acknowledgements 2](#_Toc98853765)

[Inclusion Criteria 5](#_Toc98853766)

[Exclusion Criteria 6](#_Toc98853767)

[Figure S1. All-Cause Mortality Through Day 60 in Patients with a Baseline Imputed PaO2/FiO2 ≤200. 8](#_Toc98853768)

[Figure S2. All-Cause Mortality at Day 60 for Patients Requiring High Flow Supplemental Oxygen at Baseline in Patients with a Baseline Imputed PaO2/FiO2 ≤200 9](#_Toc98853769)

[Figure S3. All-Cause Mortality at Day 60 for Patients with a Baseline Imputed PaO_2_/FiO_2_ ≤100 at Baseline 10](#_Toc98853770)

[Figure S4. Subgroup Analysis of All-Cause Mortality at Day 60 in Patients with a Baseline Imputed PaO2/FiO2 ≤200. 11](#_Toc98853771)

[Table S1: Concomitant Medications for COVID-19 in Patients with a Baseline Imputed PaO2/FiO2 ≤200 12](#_Toc98853772)

[Table S2: Baseline Characteristics (All Randomized Patients). 13](#_Toc98853773)

[Table S3. Primary and Key Secondary Endpoints (All Randomized Patients). 15](#_Toc98853774)

[Table S4: Number of Hospitalized Days or ICU Days During the First 28 Days of the Study in Patients with a Baseline Imputed PaO2/FiO2 ≤200 16](#_Toc98853775)

# Acknowledgements

We thank the patients who participated in the study and the Investigators and research teams who contributed to the study.

| **Institution** | **Investigators and Research Personnel** |
| --- | --- |
| Aurora Research Institute | Raul Mendoza-Ayala, MD, FCCP; Suraj Arora, MD; Jessica S.Thompson, AG-ACNP, MSN, BS, BSN, RN; Alexander Albers; Lori McElroe |
| Baton Rouge General Medical Center | Vasudev Tati, MD; Melissa Bunke, MD; Silpita Katragadda, MD; Jeremy Polman, DO, MS, MBA; Stacie S. LaPrarie |
| Brigham and Women's Hospital | Peter C. Hou, MD; Michelle Chung; Gabrielle Donohoe; Jordyn M. Fofi; Mohammad A. Hasdianda; Alexander B. Hill; Guruprasad Jambaulikar, MBBS, MPH; Lily Johnsky; Jenson J. Kaithamattam; Steven E. Miyawaki; Mayra A. Pinilla; Nikita Umale |
| Henry Ford Health System | Joseph Miller, MD, MS; Mayur Ramesh, MD; Hashem Nassereddine, MD; Margaret Beyer; Jo-Ann Rammal; Kathleen Wilson, RN, BSN, MS |
| JPS Health Network | James P. d'Etienne, MD, MBA; Elizabeth Cyril, MD; Stephen Q. Davis, MD, MS FCCP; Robert Peeler, MD; Maximillian Y. Masuda |
| University of Southern California | James L. Buxbaum, MD; Doug Hutcheon, MD, FHM; Jonathan Sadik, MD, Jessica Serna |
| Memorial Health Services | Maged Tanios, MD; Fady Youssef, MD; Daniel Blevins, CRC; Laura Macias |
| MaineHealth | Kathryn Brouillette, MD; David Seder, MD, FCCP, FCCM, FNCS, FAHA; Debra Burris, RN, CCRC |
| Methodist Hospital and Regions Hospital | Charles A. Bruen, MD; Michael Schnaus, MD Sadia Ali, MD; Sarah Peglow, MD; Omobosola Akinsete, MD; Firas Elmufdi, MD; Dave Niccum, MD; Sandi Wewerka, MPH; Nell Adams; Lois Akintade; Makenna Ash; Sarah Ashton; David Bachman; Ruby Carlson; Kirsten Dalrymple; Alan Denney; Jessica Haines; Miranda Harris; Hans Gil Jeong; Madison Johnson; Jenny Koops; Alana Lemke; Jennifer McKay; Meghan O’Brien; Megan O’Leary; Wiktoria Pasek; Bethany Penna; Marissa Peterson; Kelsey Schnakenberg; Jason Sloan; Zach Stoecker; Anna Swensen; Annika Tureson; Zeling Yu; Sylvia Wong; Hlee Yang; Yeshareg Yismaw |
| Houston Methodist Hospital, Department of Medicine | Mukhtar Al-Saadi, MD, EdD, FACP; Jihad G. Youssef, MD; Solmaz Afshar, Alejandro De La Torre; Anisha Gupte; Chisom Onwunyi; Caroline Perry; Mellissa Whipple; Marianne Younes |
| Northwestern University | Richard G. Wunderink, MD; Helen Donnelly, RN, BSN; Alvaro Donayre |
| Sharp Memorial Hospital | Thomas Lawrie, MD; Roland El Ghazal, MD; Cary Murphy |
| Wayne State University | James H. Paxton, MD, MBA, FACEP, FAHA; Abe Lovelace; Thomas Mazzocco; James Wooden |
| National Jewish Health | William J. Janssen, MD; Christine Griesmer, RN |
| Texas Tech University Health Sciences Center | Edward A. Michelson, MD; Danielle Austin; Lilian Marquez; Susan Watts, PhD |
| Virginia Commonwealth University | Christopher J. Hogan, MD, FACEP, FCCM; Paula Ferrada, MD, FACS; Stefan W. Leichtle, MD, FACS; Jinfeng Han, CCRP |

**Senior Research Personnel**

| Bionical-Emas | Esme Remfry; Katie Simpson, MS; Kimberly Lupejkis, BSN, RN |
| --- | --- |
| CalciMedica, Inc. | Deb Jezior; Katherine Randolph; Liisa Tingue |
| Safety Sphere | Mary Dymond BSN: Ashley Hehr; Ed Parsley, MD |

**Inclusion Criteria**

All of the following must be met for a patient to be randomized into the study:

- Has laboratory-confirmed SARS-CoV-2 infection as determined by polymerase chain reaction (PCR) or other commercial or public health assay in any specimen, as documented by either of the following:
  - PCR positive in sample collected < 72 hours prior to randomization; or
  - PCR positive in sample collected ≥ 72 hours prior to randomization, with inability to obtain a repeat sample (e.g. due to lack of testing supplies, or limited testing capacity, or results taking >24 hours, etc.) or progressive disease suggestive of ongoing SARS-CoV-2 infection;
- At least 1 of the following symptoms:
  - Fever, cough, sore throat, malaise, headache, muscle pain, dyspnea at rest or with exertion, confusion, or respiratory distress;
- At least 1 of the following signs at Screening or noted in the 24 hours before Screening:
  - PaO_2_/FiO_2_ ≤200 when receiving supplemental oxygen. The PaO_2_/FiO_2_ may be estimated from pulse oximetry or determined by arterial blood gas;
  - If SpO2 ≥97%, receiving 10L or more of supplemental oxygen;
- The presence of a respiratory infiltrate or abnormality consistent with pneumonia that is documented by either a CXR or CT scan of the lungs;
- The patient is ≥18 years of age;
- A female patient of childbearing potential must not attempt to become pregnant for 39 months, and if sexually active with a male partner, is willing to practice acceptable methods of birth control for 39 months after the last dose of study drug;
- A male patient who is sexually active with a female partner of childbearing potential is willing to practice acceptable methods of birth control for 39 months after the last dose of study drug. A male patient must not donate sperm for 39 months;
- The patient is willing and able to, or has a legal authorized representative (LAR) who is willing and able to, provide informed consent to participate, and to cooperate with all aspects of the protocol.

**Exclusion Criteria**

Patients with any of the following conditions or characteristics must be excluded from randomizing:

- Expected survival or time to withdrawal of life-sustaining treatments expected to be <7 days;
- Do Not Intubate order;
- Home mechanical ventilation (noninvasive ventilation or via tracheotomy) except for continuous positive airway pressure or bi-level positive airway pressure (CPAP/BIPAP) used solely for sleep-disordered breathing;
- PaO_2_/FiO_2_ ≤75 at the time of Screening. The PaO_2_/FiO_2_ may be estimated from pulse oximetry or determined by arterial blood gas;
- Noninvasive positive pressure ventilation;
- Invasive mechanical ventilation via endotracheal intubation or tracheostomy;
- ECMO;
- Shock defined by the use of vasopressors;
- Multiple organ dysfunction or failure;
- Positive Influenza A or B testing if tested as local standard of care;
- The patient has a history of:
  - Organ or hematologic transplant;
  - HIV
  - Active hepatitis B, or hepatitis C infection;
- Current treatment with:
  - Chemotherapy;
  - Immunosuppressive medications or immunotherapy at the time of consent;
  - Hemodialysis or Peritoneal Dialysis;
- Have a history of venous thromboembolism (VTE) (deep vein thrombosis [DVT] or pulmonary embolism [PE]) within 12 weeks prior to screening or have a history of recurrent (> 1) VTE;
- The patient is known to be pregnant or is nursing;
- Currently participating in another study of an investigational drug or therapeutic medical device at the time of consent;
- Allergy to eggs or any of the excipients in Auxora.

# Figure S1. All-Cause Mortality Through Day 60 in Patients with a Baseline Imputed PaO2/FiO2 ≤200.

The all-cause mortality rate at Day 60 was 0.21 (95% CI 0.15–0.29) for placebo and 0.12 (95% CI 0.08–0.19) for Auxora when estimated by the Kaplan-Meier procedure (*P*=0.0730). P-value is based on the stratified Kaplan-Meier estimates and standard errors estimated by Greenwood formula using the log-log transformation of the survival function stratified by the baseline imputed PaO_2_/FiO_2_ of ≤100 vs 101-200.
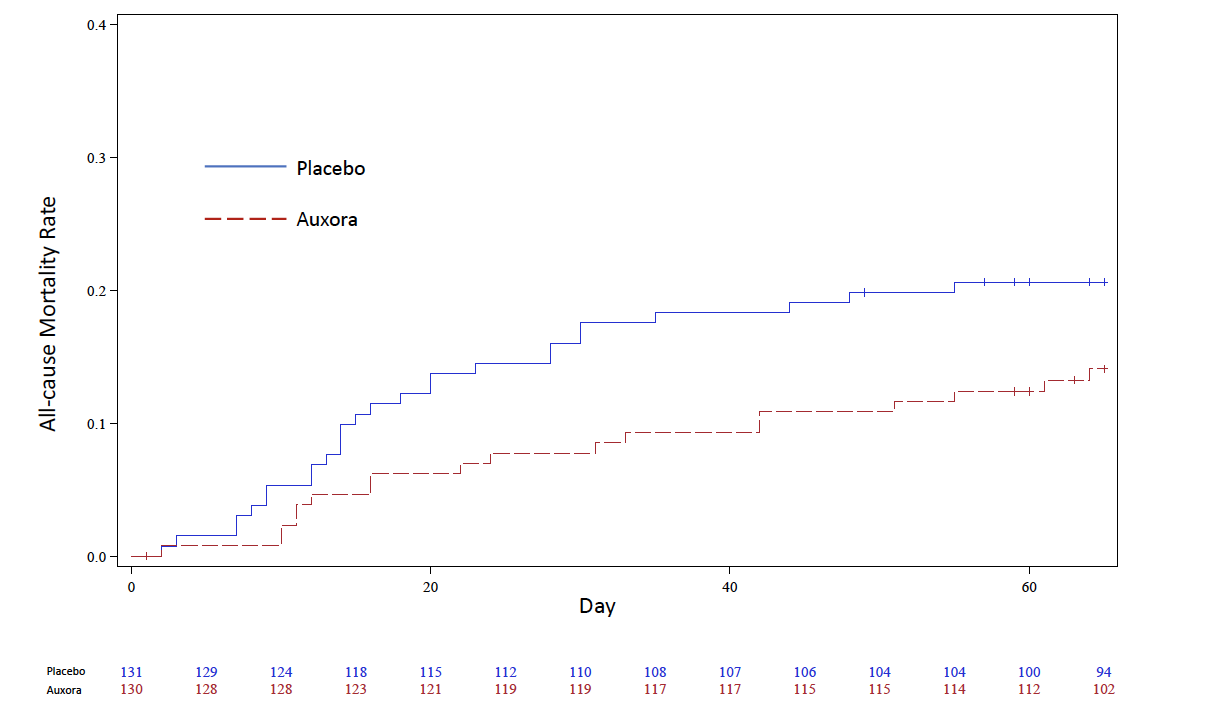


# Figure S2. All-Cause Mortality at Day 60 for Patients Requiring High Flow Supplemental Oxygen at Baseline in Patients with a Baseline Imputed PaO2/FiO2 ≤200

The all-cause mortality rate at Day 60 was 0.26 (95% CI, 0.18–0.37) for placebo and 0.15 (95% CI, 0.09–0.25) for Auxora when estimated by the Kaplan-Meier procedure (*P*=0.0980). *P*-value is based on the Kaplan-Meier estimates and standard errors estimated by Greenwood formula using the log-log transformation of the survival.

**
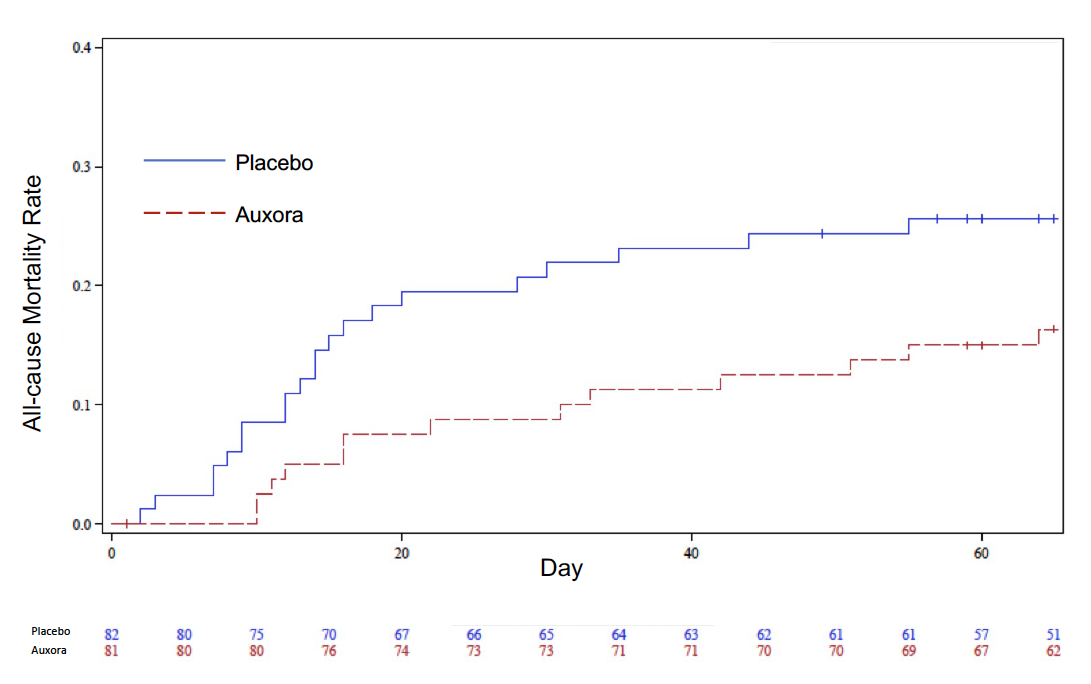
**

# Figure S3. All-Cause Mortality at Day 60 for Patients with a Baseline Imputed PaO_2_/FiO_2_ ≤100 at Baseline

The all-cause mortality rate at Day 60 was 0.29 (95% CI, 0.19–0.43) for placebo and 0.19 (95% CI, 0.11–0.32) for Auxora when estimated by the Kaplan-Meier procedure (*P*=0.1958). *P*-value is based on the Kaplan-Meier estimates and standard errors estimated by Greenwood formula using the log-log transformation of the survival.


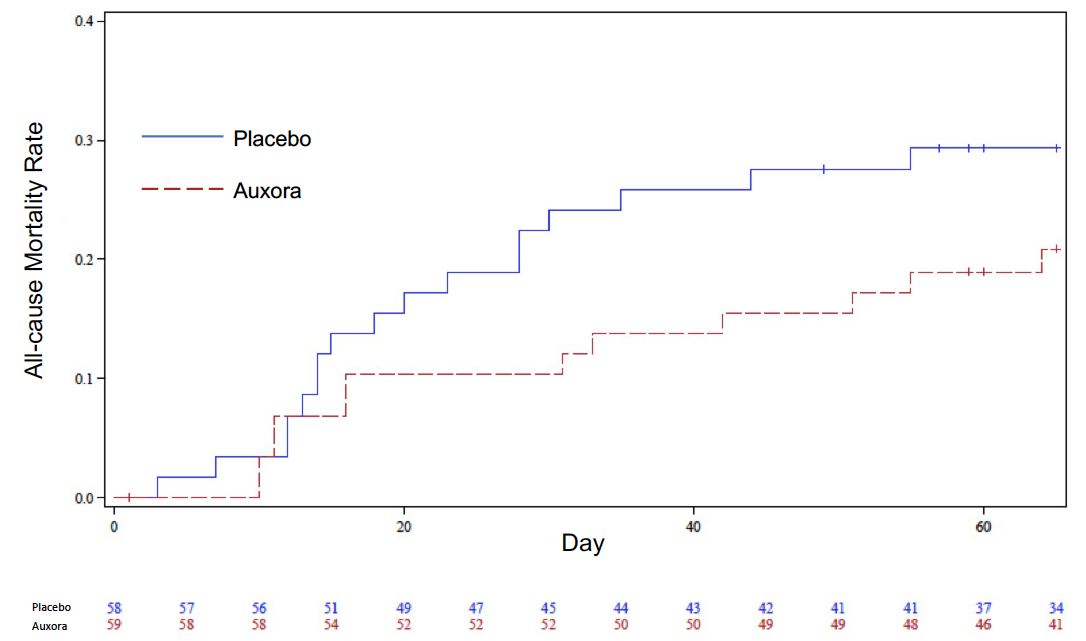


# Figure S4. Subgroup Analysis of All-Cause Mortality at Day 60 in Patients with a Baseline Imputed PaO2/FiO2 ≤200.

**
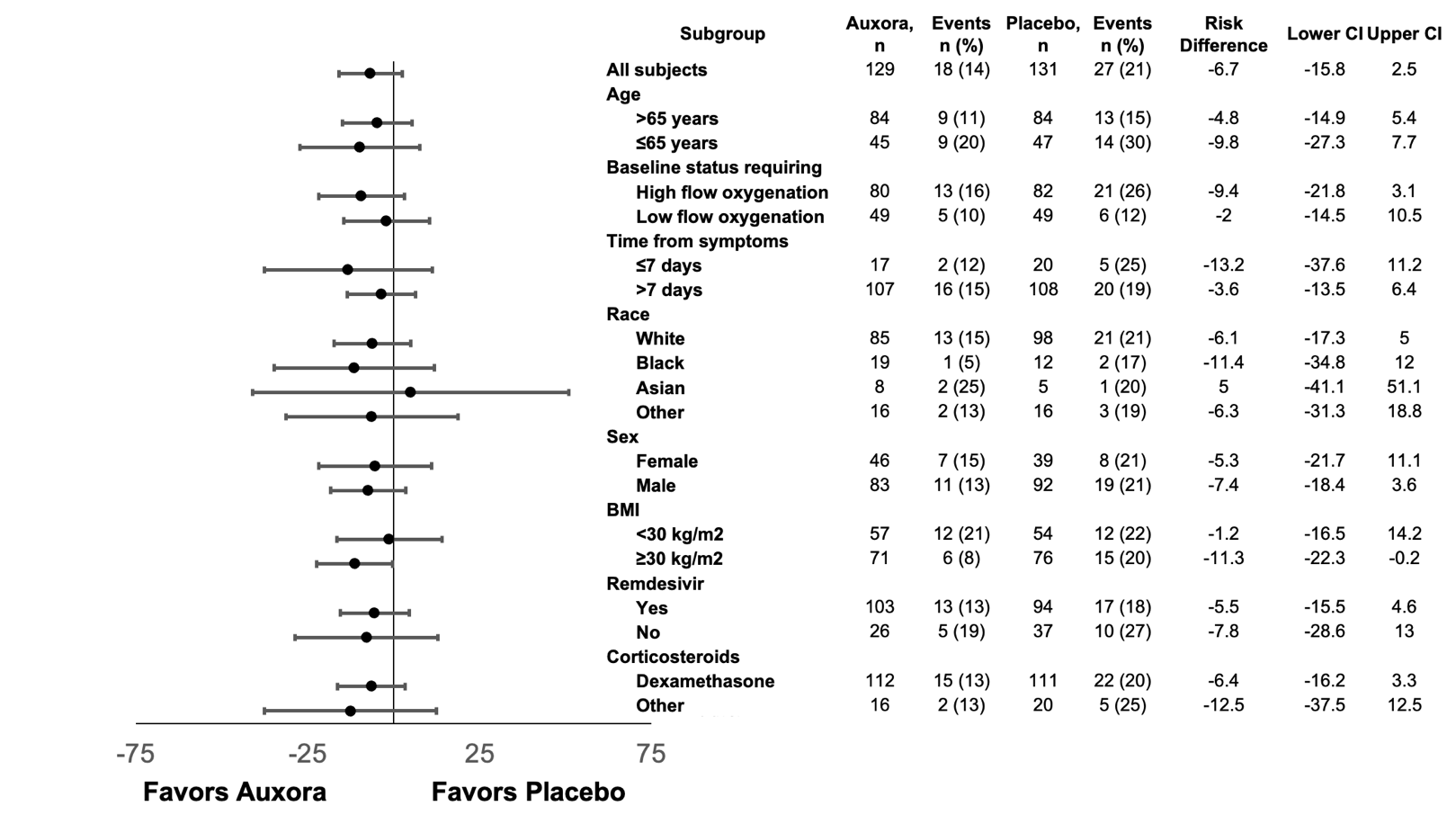
**

# Table S1: Concomitant Medications for COVID-19 in Patients with a Baseline Imputed PaO2/FiO2 ≤200

|  | Placebo  (n=131) | Auxora  (n=130) | Total  (n=261) |
| --- | --- | --- | --- |
| Corticosteroids | 131 (100%) | 130 (100%) | 261 (100%) |
| Dexamethasone | 111 (84.7%) | 113 (86.9%) | 224 (85.8%) |
| Methylprednisolone | 43 (32.8%) | 37 (28.5%) | 80 (30.7%) |
| Prednisone | 17 (13%) | 13 (10%) | 30 (11.5%) |
| Remdesivir | 94 (71.8%) | 104 (80.0%) | 198 (75.9%) |
| Anticoagulation | 130 (99.2%) | 130 (100%) | 260 (99.6%) |
| Enoxaparin | 118 (90.1%) | 125 (96.2%) | 243 (93.1%) |
| Tocilizumab | 6 (4.6%) | 2 (1.5%) | 8 (3.1%) |
| Hyperimmune Plasma COVID-19 | 2 (1.5 %) | 1 (0.8%) | 3 (1.1%) |

# Table S2: Baseline Characteristics (All Randomized Patients).

|  | Placebo  (n=141) | Auxora  (n=143) | Total  (N=284) |  |
| --- | --- | --- | --- | --- |
| Male, n (%) | 99 (70.2%) | 91 (63.6%) | 190 (66.9%) |  |
| Race |  |  |  |  |
| White, n (%) | 104 (73.8%) | 93 (65.0%) | 197 (69.4%) |  |
| Black, n (%) | 14 (9.9%) | 22 (15.4%) | 36 (12.7%) |  |
| Asian | 5 (3.5 %) | 9 (6.3 %) | 14 (4.9%) |  |
| Other/Multiple^*^ | 18 (12.8 %) | 18 (12.6 %) | 36 (12.7%) |  |
| Hispanic, n (%) | 59 (41.8%) | 49 (34.3%) | 108 (38.0%) |  |
| Mean age, years (SD) | 60.0 (12.7) | 59.1 (12.3) | 59.6 (12.5) |  |
| 65+ years of age, n (%) | 50 (35.5%) | 48 (33.6%) | 98 (34.5%) |  |
| Mean BMI, kg/m^2^ (SD) | 31.9 (6.9) | 32.9 (8.7) | 32.4 (7.8) |  |
| Mean time from symptom onset, days | 11.8 (5.8) | 11.8 (5.7) | 11.8 (5.8) |  |
| Median time from hospitalization to randomization, days | 3.0 | 3.0 | 3.0 |  |
| HFNC use, n (%) | 83 (58.9%) | 81 (56.6%) | 164 (57.7%) |  |
| Mean baseline imputed PaO_2_/FiO_2_ value^†^ (SD) | 114.7 (47.7) | 120.3 (48.6) | 117.5 (48.2) |  |
| Mean baseline imputed PaO_2_/FiO_2_ value^†^ (SD) for patients with baseline imputed PaO_2_/FiO_2_ >200 | 240.5 (29.9) | 225.4 (17.5) | 232.0 (24.3) | |
| Baseline Imputed PaO_2_/FiO_2_ ≤100, n (%) | 58 (41.1%) | 59 (41.31%) | 117 (41.2%) |  |
| Baseline Imputed PaO_2_/FiO_2_ 101-300, n (%) | 83 (58.9%) | 84 (58.7%) | 167 (58.8%) |  |
| Mean CRP, mg/L (SD) | 92.4 (67.6) | 93.8 (69.6) | 93.1 (68.5) |  |
| Mean ferritin, ng/mL (SD) | 1036.2 (856.6) | 1020.2 (946.8) | 1028.1 (901.4) |  |
| Hypertension, n (%) | 86 (61.0%) | 90 (62.9%) | 176 (62.0%) |  |
| Diabetes, n (%) | 61 (43.3%) | 60 (42.0%) | 121 (42.6%) |  |
| Hyperlipidemia, n (%) | 57 (40.4%) | 56 (39.2%) | 113 (39.8%) |  |
| Former smoker, n (%) | 36 (25.5%) | 45 (31.5%) | 81 (28.5%) |  |

^*^Other include Native Hawaiian or other Pacific Islander. One participant in the Auxora arm was missing race at baseline; ^†^Worst value in the 24 hours prior to Screening. BMI, body mass index; CRP, C-reactive protein; HFNC, high flow nasal cannula

# Table S3. Primary and Key Secondary Endpoints (All Randomized Patients).

|  | Placebo  (n=141) | Auxora  (n=143) | Difference  (95% CI) | P Value |
| --- | --- | --- | --- | --- |
| Median time to recovery, days (95% CI) | 8.0  (7.0, 11.0) | 7.0  (6.0, 8.0) |  | 0.0533 |
| All-Cause Mortality at Day 60, n (%) | 27 (19.1%) | 18 (12.6%) | -6.43  (-14.74, 1.88) | 0.1321 |

Definition of Recovery by Ordinal Scale: 6 Hospitalized, not requiring supplemental oxygen or ongoing medical care; 7 Discharged, requiring supplemental oxygen; 8 Discharged, not requiring supplemental oxygen. Analysis of time to recovery through Day 60 in the efficacy set used log-rank test stratified by the baseline imputed PaO_2_/FiO_2_ ≤100 and 101-200; Analysis of all-cause mortality in the efficacy set used Cochran-Mantel-Haenszel test stratified by the baseline imputed PaO_2_/FiO_2_ ≤100 and 101-200.

# Table S4: Number of Hospitalized Days or ICU Days During the First 28 Days of the Study in Patients with a Baseline Imputed PaO2/FiO2 ≤200

|  |  | Placebo (n=131) | Auxora  (n-130) | Difference (95% CI; P value) |
| --- | --- | --- | --- | --- |
| Hospitalized Days | LS Mean | 15.29 | 13.65 | -1.64 (-4.09–0.80; 0.1864) |
| ICU Days | LS Mean | 8.36 | 6.88 | -1.47 (-4.25–1.30; 0.2970) |

ANOVA model includes treatment group as fixed effect in the model. The number of days in the hospital is defined as 28 if the patient died. ICU, intensive care unit.
